# Association between household solid fuel usage and trajectories of multimorbidity among middle-aged and older adults: a nationwide population-based cohort study

**DOI:** 10.3389/fpubh.2024.1446688

**Published:** 2024-10-28

**Authors:** Yiting Li, Bingjie Wu, Bingbing Fan, Jiali Lv, Chunxia Li, Chang Su, Aidong Liu, Tao Zhang

**Affiliations:** ^1^Department of Biostatistics, School of Public Health, Cheeloo College of Medicine, Shandong University, Jinan, China; ^2^Institute for Medical Dataology, Cheeloo College of Medicine, Shandong University, Jinan, China; ^3^Department of Epidemiology and Biostatistics, Ministry of Education Key Lab of Environment and Health, School of Public Health, Tongji Medical College, Huazhong University of Science and Technology, Wuhan, China; ^4^Ministry of Education - Shanghai Key Laboratory of Children’s Environmental Health, Xinhua Hospital, Shanghai Jiao Tong University School of Medicine, Shanghai, China; ^5^National Institute for Nutrition and Health, Chinese Center for Disease Control and Prevention, Beijing, China

**Keywords:** air pollution, chronic diseases, longitudinal trajectory, multimorbidity, solid fuel

## Abstract

**Background:**

This study aimed to explore the effect of household solid fuel usage on the multimorbidity trajectories among middle-aged and older adults.

**Methods:**

Based on the 2011–2018 China Health and Retirement Longitudinal Study, the group-based trajectory modeling and the multinomial logistic regression model were used to explore the relationship between multimorbidity trajectories of older adults with different fuel types, duration of solid fuel usage, and potential interaction with PM_2.5_. Three multimorbidity trajectory patterns were identified by group-based trajectory modeling and labeled as “non-chronic morbidity” (no disease increase), “newly developing multimorbidity” (diseases grew from 0 to 2), and “multi-chronic multimorbidity” (diseases grew from 2 to 4).

**Results:**

Compared to “Non-chronic morbidity,” solid fuel was significantly associated with adverse multimorbidity trajectories, with an odds ratio (OR) and 95% confidence interval (CI) of 1.33 (1.11, 1.60) and 1.35 (1.18, 1.55) for newly developing and multi-chronic group, respectively. An adverse multimorbidity trajectory tended to be established with longer durations of solid fuel usage than “Non-chronic morbidity.” For “Newly-developing multimorbidity,” the ORs (95% CI) for 1–7 years and ≥ 8 years of solid fuel usage were 1.16 (0.94, 1.42) and 1.41 (1.12, 1.76), respectively, with *P* trend=0.001, while in “Multi-chronic multimorbidity,” those were 1.25 (1.07, 1.47) and 1.68 (1.41, 2.00), respectively, with *P* trend <0.001. In the interaction analysis, the association between solid fuel usage and trajectories was significant only in areas where PM_2.5_ was lower than 50 μg/m^3^.

**Conclusion:**

For the middle-aged and older Chinese population, a higher risk of multimorbidity trajectory is associated with household solid fuel usage, especially in the areas with lower PM_2.5_.

## 1 Introduction

Multimorbidity, defined as the coexistence of two or more chronic conditions, has seen a rise in the prevalence due to the global aging population (1). In China, studies of representative samples indicated that the prevalence of multimorbidity in old people is over 40% (2). Given the irreversible nature of multimorbidity, it is imperative to address its modifiable risk factors. These factors are numerous and diverse, including, but not limited to, lifestyle (3), socioeconomic status (4), and air pollution (5). Among these, both household air pollution (HAP) and outdoor air pollution (OAP) have been demonstrated to have significant correlations with multimorbidity.

The mechanisms underlying the impact of air pollution on multimorbidity are complex (2, 6). Extensive research has shown that exposure to air pollutants can affect multiple human body systems, potentially leading to a range of chronic diseases, such as hypertension, diabetes, and others (2, 7, 8). As these detrimental effects accumulate, the multimorbidity will occur. Ambient air pollution was estimated to have caused 4.2 million deaths in 2019, exceeding by over 1 million the premature deaths caused by HAP (9). However, the older population, especially those with multimorbidity, often have limited outdoor activities, which makes HAP more closely related to their health than OAP (10).

Though the studies of single air pollution on multimorbidity have existed, most of them focus on a single time point (2, 11–14). However, few research studies delve into the association of solid fuel usage and heterogeneous trajectories of chronic conditions, especially the multimorbidity trajectories. Though there are several studies of multimorbidity trajectories in foreign countries, predominantly based on the total number of chronic conditions, such as Korea, the United States, and the UK (15–20). Conversely, research studies in China on multimorbidity trajectories used a multi-trajectory model based on different disease combinations, lacking the single trajectory model (21–23). Although the multi-trajectory model may help better understand the cluster of chronic diseases, it cannot fully encompass all multimorbidity trajectories among populations. Therefore, it is imperative to conduct further research on the trajectories of multimorbidity, which is crucial for facilitating timely interventions throughout the entire lifespan. Moreover, discrepancies in chronic diseases between cooking and heating and the modified effect of PM_2.5_ on HAP should be considered (24).

Our study used four waves (2011, 2013, 2015, and 2018 years) of repeated measurement in the China Health and Retirement Longitudinal Study (CHARLS), recruiting participants aged 45 years and older to identify distinct multimorbidity trajectories. In addition, we explored the association between different types and duration of solid fuel usage and multimorbidity trajectories as well as illustrated the modifying effect of PM_2.5_ and household solid fuel usage on multimorbidity trajectories.

## 2 Materials and methods

### 2.1 Study subjects

The data for this study were obtained from CHARLS. Chaired by the National Development Research Institute of Peking University, it covered 150 counties or districts and 450 villages or resident committees of China. The baseline survey, wave 1, was conducted in 2011, and three follow-up surveys were undertaken in 2013, 2015, and 2018, respectively. The study included 45 years old and older participants, and its questionnaire was devised based on Chinese national conditions, with reference to the Health and Retirement Study, the English Longitudinal Study of Aging, and other aging survey projects in abroad. Demographic backgrounds, health status and functioning, cognition and depression, housing characteristics, and other information were collected in this study. All the questionnaires and data are publicly accessible, which can be obtained from the program website^1^ (25).

Supplementary Figure S1 shows the inclusion and exclusion process of participants in this study. First, the baseline sample of 17,705 in CHARLS was enrolled, and those lost to follow-up were excluded (*n* = 5,724). Then, we excluded those missing values on baseline fuel information (*n* = 3,054), those aging less than 45 years (*n* = 195), and those with more than two missing data on 14 chronic diseases (*n* = 328) to retain a larger sample size. After exclusion, 8,404 adult subjects, repeatedly visited four times, were included in this current study. For the analysis of the duration of fuel use, we further excluded 306 participants due to the missing fuel types for cooking and heating in waves 2013, 2015, and 2018.

### 2.2 Chronic disease and multimorbidity

During the four waves in 2011–2018, information on 14 chronic diseases was collected from respondents through two types of questions: “Have you been diagnosed with these chronic conditions by a doctor?” or “Do you know if you have these chronic conditions?” Moreover, we checked the accuracy by asking follow-up participants whether the last record was correct and whether they had been diagnosed with the conditions by a doctor since the last interview. These diseases include memory-related disease, psychiatric disorders, hypertension, dyslipidemia, diabetes, arthritis, asthma, cancer, chronic lung disease, heart attack, stroke, liver disease, digestive disease, and kidney disease. All the diseases above were defined as binary categorical variables (yes or no). For the counts of chronic conditions, we calculated the total number of chronic diseases for each participant according to a previous study (range from 0 to 14), and all of them in this study were considered as incurable illnesses (17).

### 2.3 Household fuel assessment

Information on the household fuel types was collected by asking respondents two structured questions: “What’s the main heating energy source?” and “What’s the main source of cooking fuel?,” each with seven to eight options for choosing. Types of cooking fuel were categorized into clean fuel (integrate those answering natural gas, liquefied petroleum gas, marsh gas, electric, and no cook) and solid fuel (integrate those responding coal and crop residue or wood), while heating fuel types were divided into clean fuel (integrate those answering natural gas, liquefied petroleum gas, electric, solar, and concentration heating) and solid fuel (integrate those responding coal and crop residue or wood) (26). When the respondents answered others in the above two questions, the values were marked as “missing” because of the uncertainties. In addition, we further created two variables for detail solid fuel types, whose value includes clean fuel, coal, and crop residue or wood. If participants used solid fuel for either cooking or heating, they were regarded as solid fuel users. Based on this, we defined the duration of solid fuel usage, whose values are 0 years, 1–7 years, and ≥ 8 years, corresponding to being solid fuel users in 0 waves, 1–3 waves, and 4 waves, respectively. The fuel types of combining cooking and heating were defined as both clean fuel, either solid fuel and both solid fuel 3 types.

### 2.4 PM_2.5_

The dataset of PM_2.5_ concentrations was acquired from the Atmospheric Composition Analysis Group at Dalhousie University^2^, from which the annual average PM_2.5_ concentrations of 342 administrative units in China from 2000 to 2021 were calculated (27). A linear correlation study was conducted between the recent 5-year dataset and Chinese government-published data, yielding a goodness of fit (R^2^) surpassing 0.8. Within this inquiry, the annual mean city-level PM_2.5_ concentrations from 2011 to 2018 were calculated, with each participant’s residential location linked to the corresponding PM_2.5_ data to determine their exposure window.

### 2.5 Covariates

Information for all covariates was gathered at baseline. To increase the comparability of studies, we selected all the covariates referring to a recent study (2). The covariates included demographics (including age, sex, residence, education levels, marriage status, and household annual income), lifestyle-related variables (including smoking status and drinking status), health status variables (including BMI), and environmental factors (house area, geographic position, and PM_2.5_).

### 2.6 Statistical analysis

The study population was divided into subgroups by five kinds of household fuel types (including cooking types, heating types, cooking solid fuel types, heating solid fuel types, combination of cooking and heating types) above. Moreover, we identified multimorbidity trajectories by locally weighted regression in each subgroup without considering the optimal trajectory cluster. Meanwhile, the generalized linear mixed model was applied to identify significant differences between each two curves.

Group-based trajectory modeling (GBTM) is an analysis method for longitudinal data. It assumes that there is a heterogeneity of variation among individuals in the study population and we can divide the population into several subgroups. The fixed effects between the subgroups are different (i.e., intercept and slope), while within the subgroups are the same. We can identify different subgroups whose multiple measured variables have similar changing trajectories over time through GBTM. After that, we can calculate the probability that each individual belongs to the subgroups, and we can obtain the most reasonable cluster based on it. However, the most plausible subgroups cannot be gotten before the model fitting, which should be ascertained by the following criteria after fitting the model: (1) having the smallest Bayesian information criterion (BIC); (2) average posterior probability (AvePP) > 0.70; and (3) The number of populations in each subgroup accounts for at least 5% of the whole population (28, 29). The number of chronic diseases was used to fit the trajectory using GBTM with the zero-inflated Poisson model. During the model fitting process, follow-up years were set as the time variable and were centroided (i.e., all the follow-up years used for analysis were displayed as the true value minus the mean value). The highest trajectory polynomial was carried out from linear to cubic, and we traversed one to four subgroups (for details, see Supplementary Methods 1). Finally, the optimal trajectory model was chosen from the above criteria. Among these, the definitions of multimorbidity trajectory patterns were labeled according to the initial level and trend of the trajectories, with no disease increase labeled as “Non-chronic morbidity,” diseases growing from 0 to 2 labeled as “Newly-developing multimorbidity,” and diseases growing from 2 to 4 labeled as “Multi-chronic multimorbidity.”

Multinomial logistic regression (MNL) was performed to explore the relationship of multimorbidity trajectories with four household fuel types and the duration of solid fuel usage. All the MNLs above were adjusted for confounding covariates, including baseline age, sex, residence, education level, marital status, smoking status, drinking status, BMI, and PM_2.5_. Subsequently, Z-test and stratified analyses were used to evaluate the disparities among PM_2.5_ strata. For the calculation details, see Altman et al. (30). Missing data of covariates were handled using Chained Equations (mice) 3.14.0 package (for details, see Supplementary Methods 2).

Attributed cases (AT) and population attributable fractions (PAF) were used to measure the burden of exposures to outcomes, that is, interpreting the decreasing proportion of the population with outcomes after intervening in the exposures. Our study estimated the proportion of the multimorbidity “Newly-developing multimorbidity” and “Multi-chronic multimorbidity” trajectory that might be reduced if the household solid fuel were controlled. In this study, we employed the R package AF to execute this procedure (31).

To verify the robustness of our results, sensitivity analyses were performed: (1) We stratified the analysis by the following 10 factors: age (middle-aged adults or older adults), sex (male or female individuals), BMI (< 23 kg/m^2^ or ≥ 23 kg/m^2^), residence (urban or rural), house area (≤ 120 m^2^ or > 120 m^2^), household annual income (≤ 24,000 yuan or > 24,000 yuan), an education level (incomplete compulsory education or completed compulsory education), smoking (yes or no), drinking (yes or no), and geographic position (south or north). (2) We reproduced the outcomes utilizing the data without the imputation of covariates.

The characteristics were compared using mean (SD), median [interquartile range], and *n* (percentage). In our study, except GBTM which was completed using SAS 9.4, all other analyses were performed in R 4.2.1. Hypothesis tests were two-sided, and a *p*-value of <0.05 was considered statistically significant.

## 3 Results

According to household fuel types, the baseline characteristics are presented in Supplementary Table S1. A total of 8,404 respondents were included in this study, with a median age of 57 years and slightly more female respondents (52.84%) than male respondents. Proportions of clean fuels were generally larger in cooking (*N* = 3,159, 37.59%) than in heating (*N* = 1962, 23.35%). Compared with clean fuel users, individuals using solid fuel were more likely to be older, living in rural areas, not having complete compulsory education, have less household income, be smokers, have a lower BMI, and live in smaller housing areas.

The multimorbidity trajectories in the whole population according to household fuel types indicated that the number of chronic diseases in each group all increased over time (Supplementary Figure S2). Meanwhile, the number of chronic diseases in the population with clean fuel was constantly less than those with solid fuel, whether for cooking or heating. Moreover, in the population with solid fuel usage, those using coal had a higher number of chronic diseases than those using crop residue or wood.

**Figure 1:**
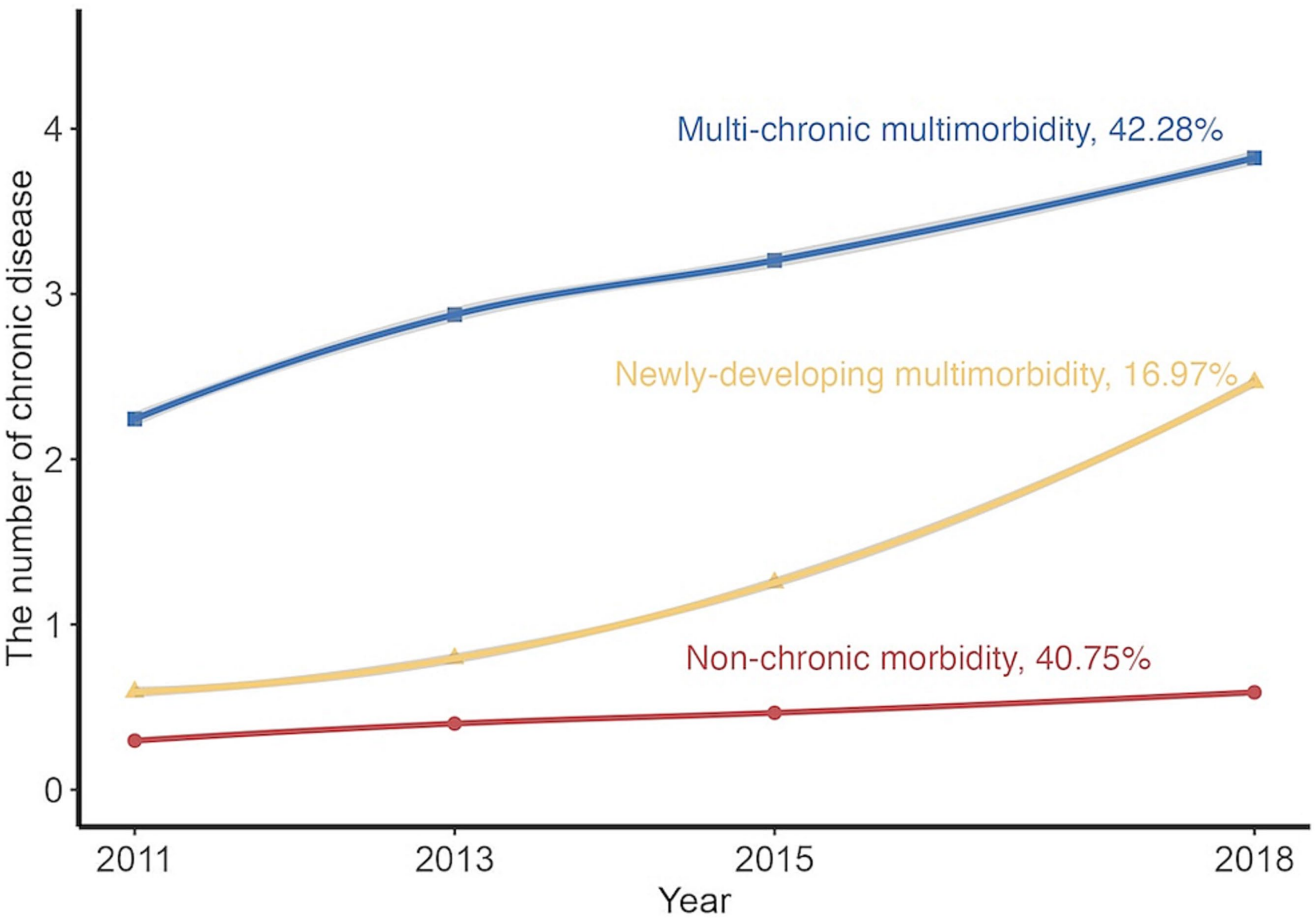
Predicted trajectories of multimorbidity from 2011 to 2018. The trajectories were shown in solid lines, and the mean of chronic disease count was expressed by solid points.

Based on the criteria mentioned above, the best-fitting model was linear trajectories of three groups, as shown in Supplementary Tables S2, S3 and Figure 1. The three groups were labeled as “Non-chronic morbidity,” “Newly-developing multimorbidity,” and “Multi-chronic multimorbidity” according to the initial level and trend of trajectories. Of these, the minority (16.40%) came from the “Newly-developing multimorbidity,” whose diseases grew from 0 to 2 on average. The “Multi-chronic multimorbidity” also presented a rising trend, with baseline diseases increasing by 2, but its trend still fell short in comparison with “Newly-developing multimorbidity.” Only the “Non-chronic morbidity” group, 41.60% of which, had no disease increase. Compared with the “Non-chronic morbidity,” participants in the multimorbidity “Newly-developing multimorbidity” or the “Multi-chronic multimorbidity” were more likely to be older adults, female, living in urban areas, with higher levels of household income, smoking less, drinking less, and having higher BMI (Table 1).

**Table 1:** Characteristics of participants by multimorbidity trajectory subgroups.

Characteristics	“Non-chronic morbidity” (*n* = 3,496)	“Newly-developing multimorbidity” (*n* = 1,378)	“Multi-chronic multimorbidity” (*n* = 3,530)
**Cooking**			
Clean fuel	1,392 (39.8)	485 (35.2)^∗^	1,282 (36.3)^∗^
Coal	431 (12.3)	192 (13.9)	464 (13.1)
Crop residue/wood	1,673 (47.9)	701 (50.9)	1784 (50.5)
**Heating**			
Clean fuel	874 (25.0)	308 (22.4)	780 (22.1)^∗^
Coal	1,301 (37.2)	545 (39.6)	1,359 (38.5)
Crop residue/wood	1,321 (37.8)	525 (38.1)	1,391 (39.4)
**Combination of cooking and heating**			
Both clean fuel	696 (19.9)	243 (17.6)^∗∗^	614 (17.4)^∗∗^
Either solid fuel	874 (25.0)	307 (22.3)	834 (23.6)
Both solid fuel	1926 (55.1)	828 (60.1)	2082 (59.0)
**Duration of solid fuel usage**			
0 years	484 (14.4)	170 (12.8)^*^	404 (11.9)^**^
1–7 years	1849 (54.9)	700 (52.5)	1745 (51.3)
≥8 years	1,032 (30.7)	463 (34.7)	1,251 (36.8)
**Age, years**	56.0 [49.8; 62.0]	57.0 [51.0; 64.0] ^∗∗^	59.0 [53.0; 65.0] ^∗∗^
**Sex**			
Male individuals	1775 (50.8)	677 (49.1)	1,511 (42.8)^∗∗^
Female individuals	1721 (49.2)	701 (50.9)	2019 (57.2)
**Residence**			
Urban	1,007 (28.8)	432 (31.3)	1,157 (32.8)^∗∗^
Rural	2,489 (71.2)	946 (68.7)	2,373 (67.2)
**Education levels**			
Incomplete compulsory education	3,134 (89.7)	1,255 (91.1)	3,202 (90.8)
Completed compulsory education	358 (10.3)	123 (8.9)	323 (9.2)
**Marriage status**			
Separated/divorced/widowed/never married	327 (9.4)	136 (9.9)	411 (11.6)
Married	3,166 (90.6)	1,242 (90.1)	3,118 (88.4)
**Household income**			
≥ average level	648 (31.6)	284 (32.3)	673 (28.4)^∗^
< average level	1,400 (68.4)	596 (67.7)	1,699 (71.6)
**Smoking status**			
Yes	1,113 (33.0)	419 (31.4)	925 (26.9)^∗∗^
No	2,258 (67.0)	915 (68.6)	2,519 (73.1)
**Drinking status**			
Yes	1,315 (37.7)	510 (37.0)	1,011 (28.7)^∗∗^
No	2,174 (62.3)	867 (63.0)	2,511 (71.3)
**BMI**			
Underweight (< 18.5kg/m^2^)	191 (6.5)	73 (6.5)^∗∗^	198 (6.8)^∗∗^
Normal weight (≥ 18.5kg/m^2^ and < 23kg/m^2^)	1,447 (49.5)	466 (41.2)	1,046 (35.7)
Overweight (≥ 23kg/m^2^ and < 25kg/m^2^)	585 (20.0)	230 (20.3)	557 (19.0)
Obese (≥25kg/m^2^)	699 (23.9)	363 (32.1)	1,127 (38.5)
**House area**			
≤120 m^2^	2,332 (67.9)	928 (68.8)	2,395 (69.2)
>120 m^2^	1,104 (32.1)	421 (31.2)	1,066 (30.8)
**Geographic position**			
South	1823 (52.1)	692 (50.2)	1827 (51.8)
North	1,673 (47.9)	686 (49.8)	1703 (48.2)
**PM**_**2.5**_ **, ug/m** ^**3**^	48.5 [37.5; 63.2]	48.5 [37.5; 63.1]	48.5 [37.2; 62.2] ^∗^

BMI, body mass index; ^∗∗^ *p* < 0.01 compared with the low-stable group; ^∗^ *p* < 0.05 compared with the low-stable group.

Table 2 displays that the usage of solid fuel was positively and significantly associated with the adverse multimorbidity trajectory (the adverse multimorbidity trajectory is referred to as the “Newly-developing multimorbidity” and the “Multi-chronic multimorbidity”). Compared with “Non-chronic morbidity,” the OR (95% CI) was 1.28 (1.11, 1.47) for cooking and 1.22 (1.04, 1.43) for heating in “Newly-developing multimorbidity,” while 1.22 (1.10, 1.36) for cooking and 1.27 (1.12, 1.43) for heating in “Multi-chronic multimorbidity.” After subdividing the type of solid fuel, this effect still remained significant. Meanwhile, we found that the longer the cumulative years of solid fuels use were, the higher risk of being an adverse multimorbidity trajectory would be, with OR (95% CI) for “Newly-developing multimorbidity” was 1.41 (1.12, 1.76) and OR (95% CI) for “Multi-chronic multimorbidity” was 1.68 (1.41, 2.00). Moreover, there was an increase in the effect with the duration of using solid fuel (*P* for trend <0.05), which also meant the clean fuel usage was protective for the multimorbidity.

**Table 2:** Relationship between different household fuel types and multimorbidity trajectories.

Characteristics	Newly-developing multimorbidity vs. Non-chronic morbidity		Multi-chronic multimorbidity vs. Non-chronic morbidity	
	OR (95% CI)	*p*-value	OR (95% CI)	*p*-value
**Cooking**				
Clean fuel	1.00 (ref)		1.00 (ref)	
Solid fuel	1.28 (1.11, 1.47)	<0.001	1.22 (1.10, 1.36)	<0.001
**Cooking solid fuel types**				
Clean fuel	1.00 (ref)		1.00 (ref)	
Coal	1.32 (1.08, 1.62)	0.008	1.20 (1.02, 1.41)	0.024
Crop residue/wood	1.27 (1.09, 1.47)	0.002	1.22 (1.09, 1.37)	<0.001
**Heating**				
Clean fuel	1.00 (ref)		1.00 (ref)	
Solid fuel	1.22 (1.04, 1.43)	0.013	1.27 (1.12, 1.43)	<0.001
**Heating solid fuel types**				
Clean fuel	1.00 (ref)		1.00 (ref)	
Coal	1.25 (1.05, 1.48)	0.012	1.26 (1.11, 1.44)	0.001
Crop residue/wood	1.19 (0.99, 1.42)	0.060	1.28 (1.11, 1.46)	<0.001
**Combination of cooking and heating**				
Both clean fuel	1.00 (ref)		1.00 (ref)	
Either solid fuel	1.05 (0.86, 1.28)	0.632	1.15 (0.99, 1.34)	0.069
Both solid fuel	1.33 (1.11, 1.60)	0.002	1.35 (1.18, 1.55)	<0.001
**Duration of solid fuel usage**				
0 year	1.00 (ref)		1.00 (ref)	
1–7 years	1.16 (0.94, 1.42)	0.165	1.25 (1.07, 1.47)	0.006
≥ 8 years	1.41 (1.12, 1.76)	0.003	1.68 (1.41, 2.00)	<0.001
***p*-value for trend**		0.001		<0.001

OR: odds ratio; CI: confidence interval; P for trend: trend test with exposure treated as an ordered variable; “Non-chronic morbidity” as reference trajectory; adjusted by baseline age, sex, residence, education level, marriage status, smoking status, drinking status, BMI, and PM_2.5_.

In Table 3, we found that 4.22% (1.69%, 6.75%) occurrence of adverse multimorbidity trajectories could be assigned to solid fuel usage for cooking. When intervening in the solid fuel for heating, there would be 5.34% (1.81%, 8.87%) populations that could avoid developing the adverse multimorbidity trajectory.

**Table 3:** Burden of “newly-developing multimorbidity” and “multi-chronic multimorbidity” caused by solid fuel.

Variable	Number of “newly-developing multimorbidity” and “multi-chronic multimorbidity”	AT (95%CI)	PAF, % (95%CI)	*p*-value
Using solid fuel for cooking (62.41%)	4,908	64 (25, 102)	4.22 (1.69, 6.75)	0.001
Using solid fuel for heating (76.65%)	4,908	81 (27, 134)	5.34 (1.81, 8.87)	0.003

AT, attributed cases; PAF, population attributable fractions.

**Figure 2:**
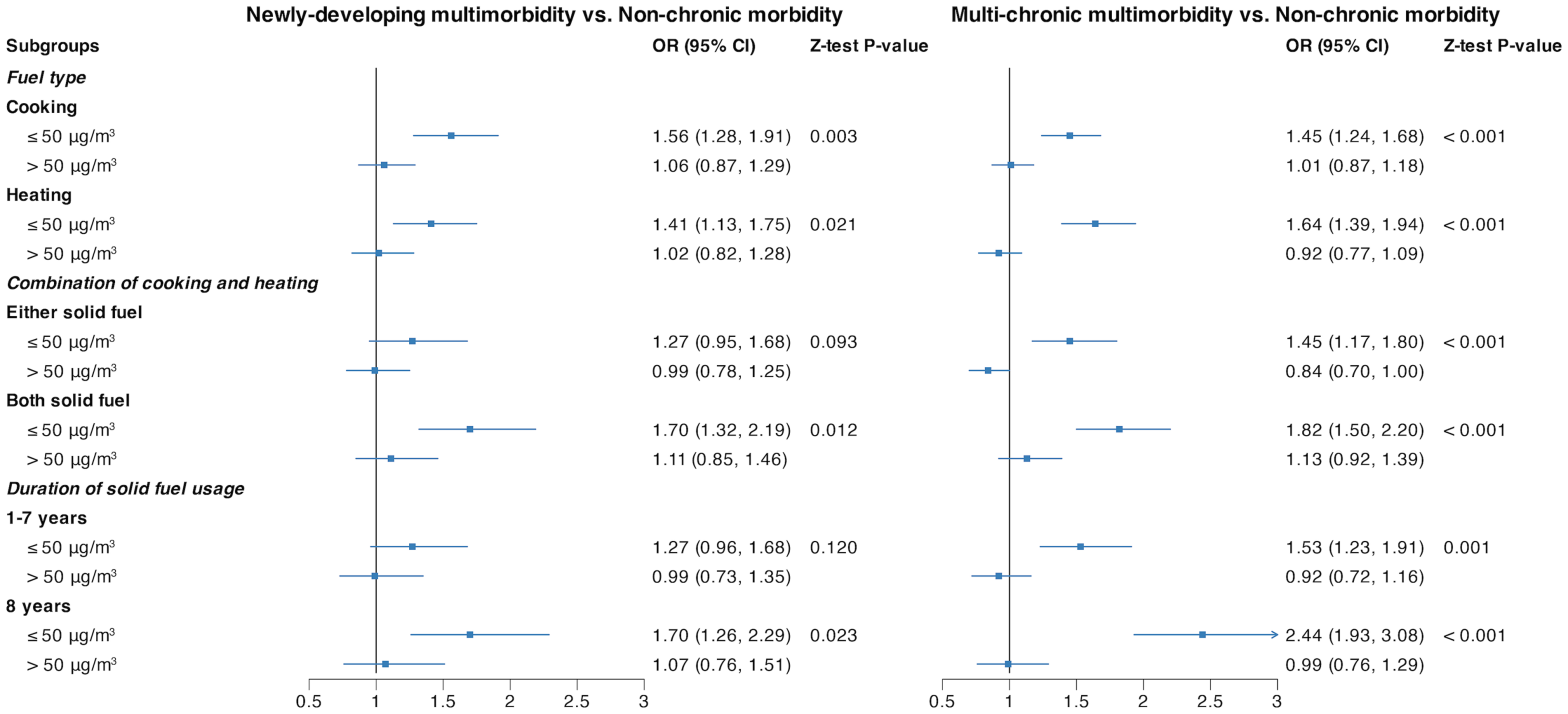
Stratified analysis by PM_2.5_ for the association of fuel types and duration of solid fuel usage with multimorbidity trajectories. Each line represents the risk of adverse multimorbidity trajectories associated with the usage of solid fuels within different PM_2.5_ subgroups. All the models were adjusted by baseline age, sex, residence, education level, marriage status, smoking status, drinking status, BMI, and PM_2.5_.

Figure 2 shows that PM_2.5_ had a marked interaction effect toward a significant association of multimorbidity trajectory with solid fuel usage. Whether using solid fuel for cooking, heating, combination, or for the duration of solid fuel usage, all scenarios exhibited a noteworthy impact in the presence of lower PM_2.5_ levels. The OR (95% CI) was 1.56 (1.28, 1.91) for cooking, 1.41 (1.13, 1.75) for heating, 1.70 (1.32, 2.19) for both solid fuel usage when combining cooking and heating, and 1.70 (1.26, 2.29) for 8 years of solid fuel usage in the context of “Newly-developing multimorbidity.” In the case of “Multi-chronic multimorbidity,” the OR (95% CI) was 1.45 (1.24, 1.68) for cooking, 1.64 (1.39, 1.94) for heating, 1.82 (1.50, 2.20) for both solid fuel usage when combining cooking and heating, and 2.44 (1.93, 3.08) for 8 years of solid fuel usage, all in comparison with the “Non-chronic morbidity” group.

Supplementary Figures S3, S4 display the results of the stratification in the sensitivity analyses. Within each subgroup, solid fuel usage remains associated with an elevated likelihood of adverse multimorbidity trajectories, few of which showed uniformity in the magnitude of solid fuel’s impact across subgroups. Individuals of a younger age, a lower BMI, a smaller residential living area, higher household annual income, incomplete compulsory education, abstention from alcohol consumption, and residing in the southern region exhibited a propensity for an augmented susceptibility to an adverse multimorbidity trajectory. The stratification in the duration of household solid fuel usage sustained the identical pattern as observed with household fuel types. Those with lower BMI, smaller residential living areas, incomplete compulsory education, and non-drinking and non-smoking habits demonstrated an elevated susceptibility. In addition, the sensitivity analysis also indicated that the results were consistent across the main analysis and the analysis without imputation (see Supplementary Tables S4, S5; Supplementary Figures S5–S7). Moreover, the inclusive population and exclusive population are generally comparable in the baseline characteristics (see Supplementary Table S6).

## 4 Discussion

Our study identified three different multimorbidity trajectories (Non-chronic morbidity, 40.75%; Newly-developing multimorbidity, 16.97%; Multi-chronic multimorbidity, 42.28%) based on a large longitudinal dataset with a nationally representative middle-aged and older adult Chinese population. Meanwhile, we found that it was easier to develop an adverse multimorbidity trajectory (i.e., “Newly-developing multimorbidity” and “Multi-chronic multimorbidity”) when using solid fuel instead of clean fuel, and the longer the duration of using solid fuel, the higher risk of being the adverse trajectory would be. Furthermore, our results suggested that ambient PM_2.5_ exposure might modify the effects of solid fuel use for heating and cooking on adverse multimorbidity trajectory risk.

Most studies identified four subgroups of multimorbidity trajectory (17, 19, 20); however, there were still few studies that identified three different multimorbidity trajectories (18), which were similar to our study. In our study, the number of chronic diseases kept zero in “Non-chronic morbidity.” Individuals in “Newly-developing multimorbidity” did not suffer any chronic conditions at baseline, but their disease number gradually increased and finally reached more than two. The highest number was observed in “Multi-chronic multimorbidity,” whose disease number increased from two to four. This also varied from the previously identified shapes of trajectories. A study of the population aged 65 years and older in Korea indicated that the majority of individuals’ multimorbidity trajectories were “maintaining-low” (59.4%), a minority of them maintained “chronically-high” (7.5%), and only two groups (“moderately-increasing,” 26.0%; “rapidly-increasing, 7.1%”) showed an increased trend over time (17). A study conducted by Tarraf et al. approximately 65 years and older Americans identified four parallel multimorbidity trajectories, and the proportions of every group were balanced (19). We speculate that this discrepancy may have come from the proportions of ethnicity, age and sex, duration of follow-up, chronic diseases selected, and indicator as a measure of multimorbidity. Currently, we only found two studies about multimorbidity trajectories in the Chinese population (21, 22), but they all used multi-trajectory modeling, which was difficult to compare with our results.

Despite previous studies only focusing on one or two cross sections, their outcomes remained consistent, in which they all found there was an increased risk of chronic disease and multimorbidity occurrence when using household solid fuels (2, 32, 33). The findings of our study were consistent with them, we found the number of chronic diseases always kept high in those using solid fuel, rather than using clean fuel whether for cooking or heating. In addition, we also found that using coal will have a higher risk of obtaining new chronic diseases than using crop residue or wood, though this remained insignificant in “Multi-chronic multimorbidity” due to some stable disease individuals in it. This may be expounded through the difference between solid biomass and coal. Compared with the solid biomass such as crop residue and wood, coal has some inherent pollutants itself, such as sulfur, arsenic, silicon dioxide, fluorine, lead, and mercury. Moreover, these pollutants would be released into the air as the original or oxidized forms in the combustion, and this would produce more pollutants (34). As a result, burning the coal has more drastically deleterious effects on health than the biomass.

Moreover, there was a study that addressed the difference in multimorbidity progression when using solid fuel (7). It found that the association between using solid fuel for heating (OR (95%CI) was 1.28 (1.09, 1.50)) and multimorbidity progression was stronger than for cooking (OR (95%CI) was 1.16 (1.01, 1.34)). Interestingly, our results did not fully align with this finding as we observed a discrepancy in the various adverse multimorbidity trajectories. Specifically, within the “Newly-developing multimorbidity” group, the risk of developing an adverse multimorbidity trajectory caused by using solid fuel for cooking was significantly higher than that for heating. Conversely, in the “Multi-chronic multimorbidity” group, the opposite outcome was observed. This variation can be attributed to two key factors. On the one hand, a higher proportion of the population used solid fuel for heating in the “Newly-developing multimorbidity” group than the “Multi-chronic multimorbidity” group, while the population using solid fuel for cooking was generally larger among the “Multi-chronic multimorbidity” group versus the “Newly-developing multimorbidity” group. On the other hand, people tend to be closer to pollution sources when cooking, thereby inhaling higher levels of pollutants than heating. However, individuals with baseline chronic diseases tended to have activity limitations, potentially leading to reduced cooking activities (35). Thus, in the “Multi-chronic multimorbidity” group, the impact of using solid fuel for heating was more pronounced, while in the “Newly-developing multimorbidity” group, the influence of using solid fuel for cooking was more significant.

In addition, our study examined whether the cumulative effect of solid fuel usage was harmful to the occurrence of adverse multimorbidity trajectory. Our results were consistent with most of the previous studies (26, 36): the longer the duration of using solid fuel, the more easily the adverse health outcome would occur. Meanwhile, in our study, this was also demonstrated by PAF. We found that reducing the usage of solid fuel could also reduce the proportion of occurring adverse health outcomes. This was in line with the findings of other studies (37, 38). Hence, one of the implications of this study is revealing the hazard of solid fuel and providing clues that changing the household fuel types (switching the solid fuel to clean fuel) could reduce the occurrence of this hazard.

Few studies linked the joint effect of HAP and OAP with multimorbidity, the majority of which only focus on the joint effect on a single disease or only investigated single air pollution. For example, a study of the UK Biobank cohort found that the risk of obtaining multimorbidity was increased by the outdoor PM_2.5_ concentrations (7). Liu et al. indicated that an obvious cumulative effect was demonstrated in solid fuel usage and exposure to PM_2.5_, coexistence of which could increase the risk of arthritis (39). Despite the available evidence showing that whether the HAP, OAP, or the joint effect of both would increase the risk of adverse health outcome occurrence, they did not distinguish whether the HAP or the OAP had different effects in different populations. Our study found that the usage of household solid fuel was the leading cause of being adverse multimorbidity trajectories among populations with lower PM_2.5_, while PM_2.5_ and other more complex reasons may be the major cause among those with higher PM_2.5_. The phenomenon in our study that individuals with solid fuel usage were more exposed to low-level ambient PM_2.5_ could corroborate this. This might be interpreted for three reasons. First, exposure to HAP has higher pollution concentrations than OAP (40). Second, individuals residing in areas with lower PM_2.5_ levels may more often disregard the implications of household air pollution (41). Furthermore, as PM_2.5_ concentrations are higher in urban areas than in rural areas (42), there existed an urban–rural disparity in the risk of HAP on multimorbidity trajectories, which is supported by the results of the stratified analyses of urban–rural differences in our study. Thus, switching from solid fuel to a cleaner alternative still needs to be strengthened in areas with lower PM_2.5_ levels and in rural areas to reduce the occurrence of adverse multimorbidity trajectories.

Previous studies suggested that there was a difference in the risk of multimorbidity when exposed to solid fuel among different populations. Zhang et al. found that male individuals had a higher risk of stroke than female individuals when using solid fuel for cooking, and smokers carried a higher risk than non-smokers as well (43). Some significant differences were found among different populations such as age, sex, BMI, residence, house area, household annual income, education level, smoking status, drinking status, and geographic position in our study, which differed from theirs. Regarding our focus on the “Newly-developing multimorbidity” group, we found intriguing outcomes in relation to residence, smoking status, and BMI, which demonstrated reversed effects during cooking and heating, though the majority of these stratified effects still underscore a consistent influence throughout cooking, heating, and amalgamation.

After stratification by residence, a higher risk of developing a “Newly-developing multimorbidity” trajectory existed in rural instead of urban when heating, which existed in urban rather than rural when cooking. Moreover, the rural exhibited a higher susceptibility to the “Newly-developing multimorbidity” risk than urban for the combination. This phenomenon was similar to that mentioned in the study undertaken by Mohajeri et al. (42), where rural areas (171 μg/m^3^, 95%CI: 153 μg/m^3^–189 μg/m^3^) showed a higher exposure of household solid fuel usage for combination than urban areas (92 μg/m^3^, 95%CI: 77 μg/m^3^–106 μg/m^3^). The discrepancy between rural and urban areas might be attributed to the lower quality of heating, such as heating stoves or “kang,” being more common in rural settings, while radiators or air conditioners are prevalent in urban areas (44). In contrast, when it comes to cooking, rural areas tend to conduct this activity outdoors, whereas urban areas do not (45). As described in previous studies (46, 47), the presence of environmental pollutants exacerbates health problems caused by smoking. Consequently, smokers may have a higher risk of developing adverse multimorbidity trajectories, whether using solid fuel for cooking or heating. However, the observation of higher risk in non-smokers when cooking in this study may be because some smokers may lack cooking habits. However, the reason for the diversity in different BMI populations still remains unclear. Cao et al. suggested that long-term air pollution might elevate adipose lipolysis, thereby contributing to the initiation of oxidative stress and inflammation pathways (48). In addition, longer periods of pollutant exposure were demonstrated to exist in populations using solid fuel for heating rather than for cooking (49). Therefore, it is reasonable to assume that individuals with lower BMIs may have a higher risk of developing multimorbidity trajectories than those with higher BMIs when heating, while the effect may be the opposite when cooking.

This study had some limitations. First, similar to another study (50), we did not obtain the indoor pollutants’ concentrations, just replaced this by using self-reported household fuel types, which may neglect the effect of some factors such as ventilation and house types on HAP. Second, the lack of measurement on cooking frequency limits the ability to obtain more precise individual exposure to pollutants from cooking, as relying solely on whether one cooks or not is imprecise. This still needs further attention. Third, the chronic disease information was collected by self-reported, which was not as accurate as medical records and remained some information bias. Fourth, in our studies, only fine particulate matter (PM_2.5_) among OAP was taken into account. The remaining indicators of OAP still warrant subsequent study. Furthermore, there still existed some confounding factors not considered, which could also cause effects on the occurrence of chronic disease in the study population, such as household income. Thus, their real relationship could be exaggerated or masked. Finally, as with all cohorts, the selection bias caused by loss to the follow-up was not negligible.

## 5 Conclusion

This study identified three different multimorbidity trajectories and found that household solid fuel usage increases the risk of adverse multimorbidity trajectories. Moreover, the modification of PM_2.5_ in the effects of household solid fuel use on multimorbidity trajectories existed, and those living with lower PM_2.5_ are susceptible to solid fuel usage. Our study highlights the important implications for reducing the long-term burden of multimorbidity by switching to cleaner fuels.

## Data Availability

The original contributions presented in the study are included in the article/Supplementary material, further inquiries can be directed to the corresponding authors.

## Footnotes

^1^ http://charls.pku.edu.cn/

^2^ https://sites.wustl.edu/acag/datasets/surface-pm2-5/
