# Supplementary material for: Association between household solid fuel usage and trajectories of multimorbidity among middle-aged and older adults: a nationwide population-based cohort study
Source: Front Public Health. 2024 Oct 28;12:1446688. doi: 10.3389/fpubh.2024.1446688 (PMC11551997; doi:10.3389/fpubh.2024.1446688)
Supplement: Supplementary file 1 [file Data_Sheet_1.docx]

***Supplemental Materials***

**Association between solid fuel usage and multimorbidity trajectories in middle-aged and elderly people: a nationwide population-based cohort study**

**Table of contents**

**Section 1 Supplementary Methods**

***Supplementary Method 1.*** *Fitting process of group-based trajectory modeling*

***Supplementary Method 2.*** *Imputation methods of covariate in this study*

**Section 2 Supplementary Tables**

***Table S1.*** *Baseline characteristics by household fuel types.*

***Table S2.*** *Group-based trajectory modeling (GBTM) results of model fitting process for multimorbidity.*

***Table S3****. Parameters estimates for the best fitting 4 groups GBTM for multimorbidity.*

***Table S4.*** *The relationship between different household fuel types and multimorbidity trajectories before imputation.*

***Table S5.*** *The burden of "Newly-developing multimorbidity" and "Multi-chronic multimorbidity" caused by soli fuel before imputation.*

***Table S6.*** *Characteristics of the study population and the population excluded.*

**Section 3 Supplementary Figures**

***Figure S1.*** *Flowchart of analytic sample selection.*

***Figure S2.*** *The overall multimorbidity trajectories in different household fuel types.*

***Figure S3.*** *The association between different household fuel use and multimorbidity. trajectories after stratified.*

***Figure S4****. The association between solid fuel using duration and multimorbidity trajectories after stratified.*

***Figure S5.*** *Stratified analysis by PM_2.5_ for the association of fuel types and duration of solid fuel usage with multimorbidity trajectories before imputation.*

***Figure S6.*** *The association between different household fuel use and multimorbidity trajectories after stratified before imputation.*

***Figure S7.*** *The association between solid fuel using duration and multimorbidity trajectories after stratified before imputation.*

**Section 1 Supplementary Methods**

**Supplementary Method 1. Fitting process of group-based trajectory modeling:**

We utilized group-based trajectory modeling (GBTM) to investigate distinct multimorbidity trajectories among middle-aged and older adults. Data preparation, modeling, and visualizations were carried out using SAS (version 9.4) and R (version 4.2.1).

GBTM implementation involves iterative procedures and requires a priori decisions informed by field-specific knowledge and statistical inference (1). To better illustrate the implementation process, we summarize the GBTM fitting process for this study in the following four steps:

**- Step 1: Problem Definition and Determination of Trajectory Subgroups**

We hypothesized that the number of chronic diseases among middle-aged and older adults follows heterogeneous trajectories. Considering that multimorbidity trajectories in China tend to exhibit multiple trajectories rather than a single trajectory, and that most single multimorbidity trajectories in foreign countries comprise three or four groups, we assumed that the number of chronic disease trajectories would be three or four groups.

**- Step 2: Model Specification**

During the fitting of the GBTM model, we evaluated polynomial functions including linear, quadratic, and cubic, and tested grouping numbers from 1 to 4 within each function form.

**- Step 3: Model Estimation**

The models were estimated using Bayesian methods.

**- Step 4: Model Selection and Interpretation**

The goal of this step was to determine which of the tested models provides the best or most reasonable representation of the observed data. We selected the optimal model fit based on the following criteria:

(1) High mean posterior probabilities (≥ 0.7).

(2) An acceptable proportion of the population (≥ 5.0%).

(3) The lowest Bayesian information criterion.

(4) Prior knowledge of the research.

**Supplementary Method 2. Imputation methods of covariate in this study:**

In this study, we employed the Multivariate Imputation by Chained Equations (MICE) method for covariate imputation (2). This method is based on the assumption of Missing Completely At Random (MCAR) or Missing At Random (MAR) patterns. All the selected covariates were assumed to follow a MAR pattern, indicating that the missing data is random but may be related to other observed variables.

Before the imputation process, we assessed the amount of missing data for each selected variable (3). Any variable with more than 20% missing values was excluded. For instance, the proportion of missing data for exercise was 60%, and for MMSE score, it was 37%, so they were excluded. Consequently, we selected the following baseline covariates: age, sex, residence, education level, marital status, smoking status, drinking status, BMI, and PM_2.5_.

During the imputation process, all the selected baseline covariates were included in the model. We utilized the 'mice' package (version 3.16.0) and set the imputation method to 'cart' for handling missing data. The number of multiple imputations was set to 10. In the MICE algorithm, 10 different imputed results were generated for each variable requiring imputation. For continuous variables, the final imputed values were set as the mean of the 10 imputed results. For categorical variables, the final imputed values were set to those with a frequency of more than five.

**References**

1. Nagin DS, Jones BL, Passos VL, Tremblay RE. Group-Based Multi-Trajectory Modeling. *Stat Methods Med Res* (2018) 27(7):2015-23. Epub 20161017. doi: 10.1177/0962280216673085.

2. Buuren Sv, Groothuis-Oudshoorn K. Mice: Multivariate Imputation by Chained Equations Inr. *Journal of Statistical Software* (2011) 45(3). doi: 10.18637/jss.v045.i03.

3. Shi W, Zhang T, Li Y, Huang Y, Luo L. Association between Household Air Pollution from Solid Fuel Use and Risk of Chronic Diseases and Their Multimorbidity among Chinese Adults. *Environ Int* (2022) 170:107635. Epub 20221115. doi: 10.1016/j.envint.2022.107635.

**Section 2 Supplementary Tables**

**Table S1.** Baseline characteristics by household fuel types

| **Characteristics** | **Cooking** | | **Heating** | |
| --- | --- | --- | --- | --- |
|  | **Clean fuel (N=3159)** | **Solid fuel (N=5245)** | **Clean fuel (N=1962)** | **Solid fuel (N=6442)** |
| **Age, years** | 56.0 [50.0; 62.0] | 58.0 [52.0; 64.0]^**^ | 56.0 [50.0; 63.0] | 58.0 [51.0; 64.0]^**^ |
| **Sex** |  |  |  |  |
| Male | 1523 (48.2) | 2440 (46.5) | 938 (47.8) | 3025 (47.0) |
| Female | 1636 (51.8) | 2805 (53.5) | 1024 (52.2) | 3417 (53.0) |
| **Residence** |  |  |  |  |
| Urban | 1574 (49.8) | 1022 (19.5)^**^ | 1093 (55.7) | 1503 (23.3)^**^ |
| Rural | 1585 (50.2) | 4223 (80.5) | 869 (44.3) | 4939 (76.7) |
| **Education levels** |  |  |  |  |
| Incomplete compulsory education | 2668 (84.6) | 4923 (93.9)^**^ | 1660 (84.7) | 5931 (92.2)^**^ |
| Completed compulsory education | 486 (15.4) | 318 (6.07) | 299 (15.3) | 505 (7.85) |
| **Marriage status** |  |  |  |  |
| Separated/divorced/widowed/never married | 308 (9.76) | 566 (10.8) | 189 (9.63) | 685 (10.6) |
| Married | 2849 (90.2) | 4677 (89.2) | 1773 (90.4) | 5753 (89.4) |
| **Household income** |  |  |  |  |
| ≥ average level | 804 (45.3) | 801 (22.7)^**^ | 555 (53.6) | 1050 (24.6)^**^ |
| < average level | 969 (54.7) | 2726 (77.3) | 480 (46.4) | 3215 (75.4) |
| **Smoking status** |  |  |  |  |
| Yes | 888 (28.9) | 1569 (30.9)^*^ | 541 (28.3) | 1916 (30.7)^*^ |
| No | 2180 (71.1) | 3512 (69.1) | 1371 (71.7) | 4321 (69.3) |
| **Drinking status** |  |  |  |  |
| Yes | 1114 (35.3) | 1722 (32.9)^*^ | 696 (35.5) | 2140 (33.3) |
| No | 2038 (64.7) | 3514 (67.1) | 1265 (64.5) | 4287 (66.7) |
| **BMI** |  |  |  |  |
| Underweight (< 18.5) | 133 (5.2) | 329 (7.4)^**^ | 66 (4.2) | 396 (7.3)^**^ |
| Normal weight (≥ 18.5 and < 23) | 981 (38.6) | 1978 (44.5) | 635 (40.4) | 2324 (42.9) |
| Overweight (≥ 23 and < 25) | 528 (20.8) | 844 (19.0) | 342 (21.8) | 1030 (19.0) |
| Obese (≥ 25) | 900 (35.4) | 1289 (29.0) | 527 (33.6) | 1662 (30.7) |
| **House area** |  |  |  |  |
| ≤ 120 m^2^ | 2014 (64.9) | 3641 (70.8)^**^ | 1212 (62.8) | 4443 (70.3)^**^ |
| > 120 m^2^ | 1090 (35.1) | 1501 (29.2) | 718 (37.2) | 1873 (29.7) |

**Table S1.** Baseline characteristics by household fuel types (continued)

| **Characteristics** | **Cooking** | | **Heating** | |
| --- | --- | --- | --- | --- |
|  | **Clean fuel (N=3159)** | **Solid fuel (N=5245)** | **Clean fuel (N=1962)** | **Solid fuel (N=6442)** |
| **Geographic position** |  |  |  |  |
| South | 1925 (60.9) | 2417 (46.1)^**^ | 1661 (84.7) | 2681 (41.6)^**^ |
| North | 1234 (39.1) | 2828 (53.9) | 301 (15.3) | 3761 (58.4) |
| **PM_2.5_, µg/m^3^** | 50.6 [37.6; 63.2] | 48.2 [37.2; 62.2]^**^ | 48.2 [37.2; 56.1] | 48.5 [37.5; 63.5]^**^ |

*Note*. BMI, body mass index. ^∗∗^ indicates *P* < 0.01; ^∗^ indicates *P* < 0.05.

**Table S2.** Group-based trajectory modeling (GBTM) results of model fitting process for multimorbidity

| **Group** | **Polynomial degree** | **Log-Lik** | **BIC1** | **BIC2** | **Group percentage (%)** | **Mean posterior probabilities (%)** |
| --- | --- | --- | --- | --- | --- | --- |
| 1 | 1 | -58991.095 | -59000.131 | -59001.518 | 100 | 1 |
| 1 | 2 | -58969.964 | -58983.519 | -58985.598 | 100 | 1 |
| 1 | 3 | -58961.607 | -58979.68 | -58982.453 | 100 | 1 |
| 2 | 0 1 | -40609.511 | -40627.584 | -40630.356 | 48.74/51.26 | 0.98/0.99 |
| 2 | 0 2 | -40555.828 | -40578.42 | -40581.885 | 48.89/51.11 | 0.98/0.99 |
| 2 | 0 3 | -40551.505 | -40578.615 | -40582.773 | 48.88/51.12 | 0.98/0.99 |
| **3** | **0 1 1** | **-36503.905** | **-36535.533** | **-36540.385** | **40.75/16.97/42.28** | **0.98/0.96/0.99** |
| 3 | 1 2 2 | -46311.691 | -46356.874 | -46363.805 | 22.86/53.32/23.82 | 0.96/0.93/0.9 |
| 3 | 1 3 3 | -46305.834 | -46360.053 | -46368.371 | 22.88/53.32/23.81 | 0.96/0.93/0.9 |
| 4 | 0 1 1 1 | -46462.123 | -46507.306 | -46514.237 | 16.21/40/36.28/7.51 | 0.95/0.9/0.84/0.83 |
| 4 | 0 1 2 2 | -46449.987 | -46504.205 | -46512.523 | 16.21/39.92/36.34/7.54 | 0.95/0.9/0.84/0.83 |
| 4 | 0 3 3 2 | -46442.311 | -46510.085 | -46520.482 | 16.21/40.01/36.31/7.47 | 0.95/0.9/0.84/0.83 |

*Note*. Log-Lik: the maximum Log-Likelihood; BIC1: the Bayesian information Criterion based on observations; BIC2: the Bayesian information Criterion based on individuals; The best fitting model is highlighted in bold characters.

**Table S3.** Parameters estimates for the best fitting 4 groups GBTM for multimorbidity

| **Trajectory groups** | **Polynomial degree** | **β** | **SD** | **T for H0: parameter** | ***P*-value** |
| --- | --- | --- | --- | --- | --- |
| 1 | Intercept | -3.197 | 0.091 | -35.117 | < 0.001 |
| 2 | Intercept | 0.213 | 0.013 | 16.231 | < 0.001 |
| 2 | Linear | 0.112 | 0.003 | 42.168 | < 0.001 |
| 3 | Intercept | 1.239 | 0.010 | 129.201 | < 0.001 |
| 3 | Linear | 0.071 | 0.002 | 32.530 | < 0.001 |

**Table S4.** The relationship between different household fuel types and multimorbidity trajectories before imputation

| **Characteristics** | **Newly-developing multimorbidity vs. Non-chronic morbidity** | | **Multi-chronic multimorbidity vs. Non-chronic morbidity** | |
| --- | --- | --- | --- | --- |
|  | **OR (95% CI)** | ***P*-value** | **OR (95% CI)** | ***P*-value** |
| **Cooking** |  |  |  |  |
| Clean fuel | 1.00 (ref) |  | 1.00 (ref) |  |
| Solid fuel | 1.28 (1.10, 1.49) | 0.002 | 1.17 (1.04, 1.31) | 0.008 |
| **Cooking solid fuel types** |  |  |  |  |
| Clean fuel | 1.00 (ref) |  | 1.00 (ref) |  |
| Coal | 1.36 (1.09, 1.70) | 0.006 | 1.16 (0.97, 1.37) | 0.097 |
| Crop residue/ wood | 1.25 (1.06, 1.47) | 0.007 | 1.18 (1.04, 1.33) | 0.010 |
| **Heating** |  |  |  |  |
| Clean fuel | 1.00 (ref) |  | 1.00 (ref) |  |
| Solid fuel | 1.21 (1.02, 1.44) | 0.032 | 1.22 (1.07, 1.40) | 0.003 |
| **Heating solid fuel types** |  |  |  |  |
| Clean fuel | 1.00 (ref) |  | 1.00 (ref) |  |
| Coal | 1.24 (1.02, 1.50) | 0.030 | 1.23 (1.06, 1.42) | 0.005 |
| Crop residue/ wood | 1.18 (0.97, 1.44) | 0.097 | 1.22 (1.05, 1.41) | 0.010 |
| **Combination of cooking and heating** |  |  |  |  |
| Both clean fuel | 1.00 (ref) |  | 1.00 (ref) |  |
| Either solid fuel | 1.09 (0.87, 1.37) | 0.450 | 1.18 (1.00, 1.40) | 0.049 |
| Both solid fuel | 1.34 (1.10, 1.65) | 0.004 | 1.30 (1.12, 1.52) | 0.001 |
| **Duration of solid fuel use** |  |  |  |  |
| 0 year | 1.00 (ref) |  | 1.00 (ref) |  |
| 1-7 years | 1.21 (0.95, 1.53) | 0.117 | 1.30 (1.08, 1.56) | 0.005 |
| ≥ 8 years | 1.39 (1.08, 1.80) | 0.012 | 1.62 (1.33, 1.98) | <0.001 |
| ***P*-value for trend** |  | 0.009 |  | <0.001 |

*Note*. OR: odds ratio; CI: confidence interval; *P* for trend: trend test with exposure treated as an ordered variable; “Non-chronic morbidity” as reference trajectory; Adjusted by baseline age, sex, residence, education level, marriage status, smoking status, drinking status, BMI, PM_2.5_.

**Table S5.** The burden of "Newly-developing multimorbidity" and "Multi-chronic multimorbidity" caused by soli fuel before imputation

| Variable | Number of "Newly-developing multimorbidity" and "Multi-chronic multimorbidity" | AT (95%CI) | PAF, % (95%CI) | *P*-value |
| --- | --- | --- | --- | --- |
| Using solid fuel for cooking (62.41%) | 4908 | 230 (95, 366) | 4.70 (1.94, 7.47) | <0.001 |
| Using solid fuel for heating (76.65%) | 4908 | 205 (116, 495) | 6.23 (2.38, 10.09) | 0.002 |

*Note*. AT: attributed cases; PAF: population attributable fractions.**Table S6.** Characteristics of the study population and the population excluded

| **Characteristics** | **The study population (N=8408)** | **The population excluded (N=9301)** | **The total population in 2011 (N=17705)** |
| --- | --- | --- | --- |
| **Cooking** |  |  |  |
| Clean fuel | 3159 (37.59) | 4820 (53.85) | 7979 (45.98) |
| Coal | 1087 (12.93) | 738 (8.25) | 1825 (10.52) |
| Crop residue/ wood | 4158 (49.48) | 3392 (37.90) | 7550 (43.51) |
| **Heating** |  |  |  |
| Clean fuel | 1962 (23.35) | 1398 (31.65) | 3360 (26.21) |
| Coal | 3205 (38.14) | 1448 (32.78) | 4653 (36.29) |
| Crop residue/ wood | 3237 (38.52) | 1571 (35.57) | 4808 (37.50) |
| **Combination of cooking and heating** |  |  |  |
| Both clean fuel | 1553 (18.48) | 1185 (26.96) | 2738 (21.39) |
| Either solid fuel | 4836 (57.54) | 2250 (51.18) | 7086 (55.36) |
| Both solid fuel | 2015 (23.98) | 961 (21.86) | 2976 (23.25) |
| **Age, years** | 57 [51, 64] | 58 [50, 67] | 58 [51, 65] |
| **Sex** |  |  |  |
| Male | 3963 (47.16) | 4514 (48.54) | 8477 (47.88) |
| Female | 4441 (52.84) | 4785 (51.46) | 9226 (52.12) |
| **Residence** |  |  |  |
| Urban | 2596 (30.89) | 4572 (49.16) | 7168 (40.49) |
| Rural | 5808 (69.11) | 4729 (50.84) | 10537 (59.51) |
| **Education levels** |  |  |  |
| Incomplete compulsory education | 7591 (90.42) | 7816 (84.42) | 15407 (87.28) |
| Completed compulsory education | 804 (9.58) | 1442 (15.58) | 2246 (12.72) |
| **Marriage status** |  |  |  |
| Separated/divorced/widowed/never married | 874 (10.40) | 1384 (14.92) | 2258 (12.78) |
| Married | 7526 (89.60) | 7891 (85.08) | 15417 (87.22) |
| **Household income** |  |  |  |
| ≥ average level | 1605 (30.28) | 2010 (38.35) | 3615 (34.29) |
| < average level | 3695 (69.72) | 3231 (61.65) | 6926 (65.71) |
| **Smoking status** |  |  |  |
| Yes | 2457 (30.15) | 2405 (27.47) | 4862 (28.76) |
| No | 5692 (69.85) | 6351 (72.53) | 12043 (71.24) |
| **Drinking status** |  |  |  |
| Yes | 2836 (33.81) | 2931 (32.02) | 5767 (32.88) |
| No | 5552 (66.19) | 6222 (67.98) | 11774 (67.12) |
| **BMI** |  |  |  |
| Underweight (< 18.5) | 462 (6.62) | 487 (7.34) | 949 (6.97) |
| Normal weight (≥ 18.5 and < 23) | 2959 (42.38) | 2758 (41.54) | 5717 (41.97) |

**Table S6.** Characteristics of the study population and the population excluded (continued)

| **Characteristics** | **The study population (N=8408)** | **The population excluded (N=9301)** | **The total population in 2011 (N=17705)** |
| --- | --- | --- | --- |
| Overweight (≥ 23 and < 25) | 1372 (19.65) | 1385 (20.86) | 2757 (20.24) |
| Obese (≥ 25) | 2189 (31.35) | 2009 (30.26) | 4198 (30.82) |
| **House area** |  |  |  |
| ≤ 120 m^2^ | 5655 (68.58) | 6353 (72.07) | 12008 (70.38) |
| > 120 m^2^ | 2591 (31.42) | 2462 (27.93) | 5053 (29.62) |
| **Geographic position** |  |  |  |
| South | 4342 (51.67) | 5524 (59.99) | 9866 (56.02) |
| North | 4062 (48.33) | 3684 (40.01) | 7746 (43.98) |
| **PM_2.5_, µg/m^3^** | 48.49 [37.48, 62.53] | 48.16 [37.02, 59.24] | 48.2 [37.22, 60.12] |

*Note*. Continuous numeric variables are expressed as Median [IQR], categorical variables are expressed as frequency (percentage); Using ANOVA or Kruskal-Wallis H test for continuous numeric variables and chis-square test for categorical variables.

**Section 3 Supplementary Figures**


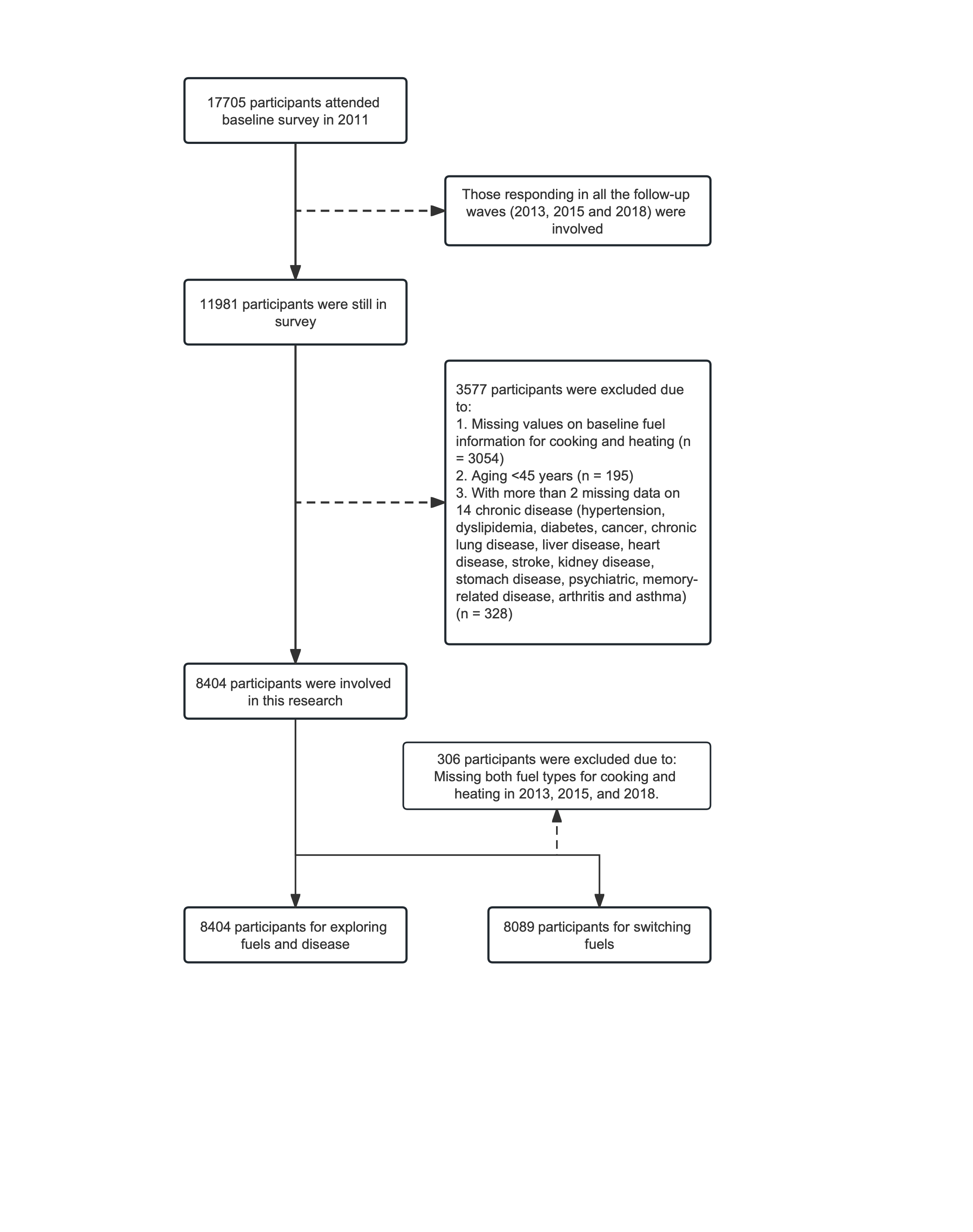


**Figure S1.** Flowchart of analytic sample selection


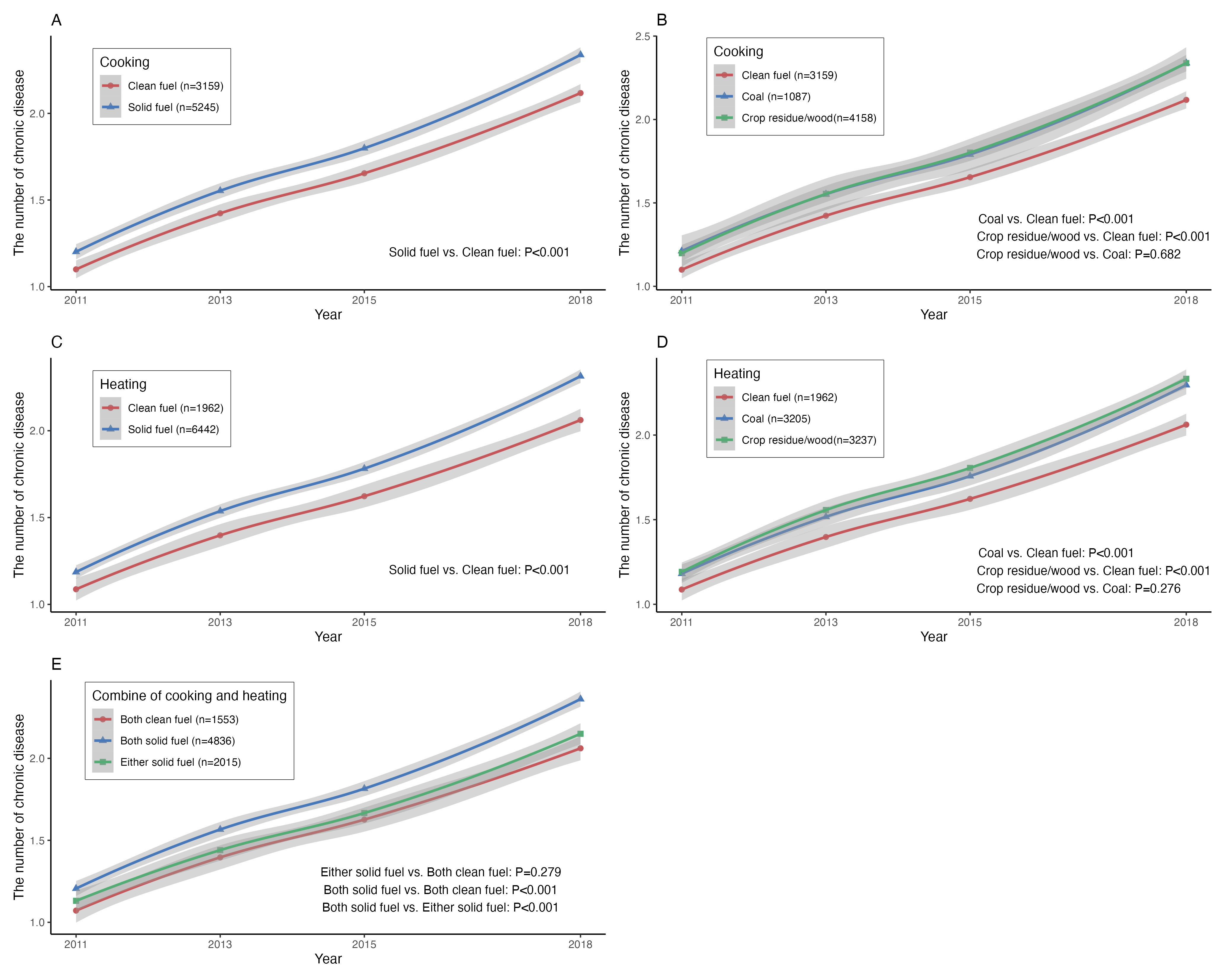


**Figure S2.** The overall multimorbidity trajectories in different household fuel types

*P* was for the interaction of fuel types and wave. A: the total population was divided into cooking clean fuel group and cooking solid group; B: the total population was divided into cooking clean fuel group, cooking coal group and cooking crop residue or wood group; C: the total population was divided into cooking clean fuel group and cooking solid group; D: the total population was divided into cooking clean fuel group, cooking coal group and cooking crop residue or wood group.

**
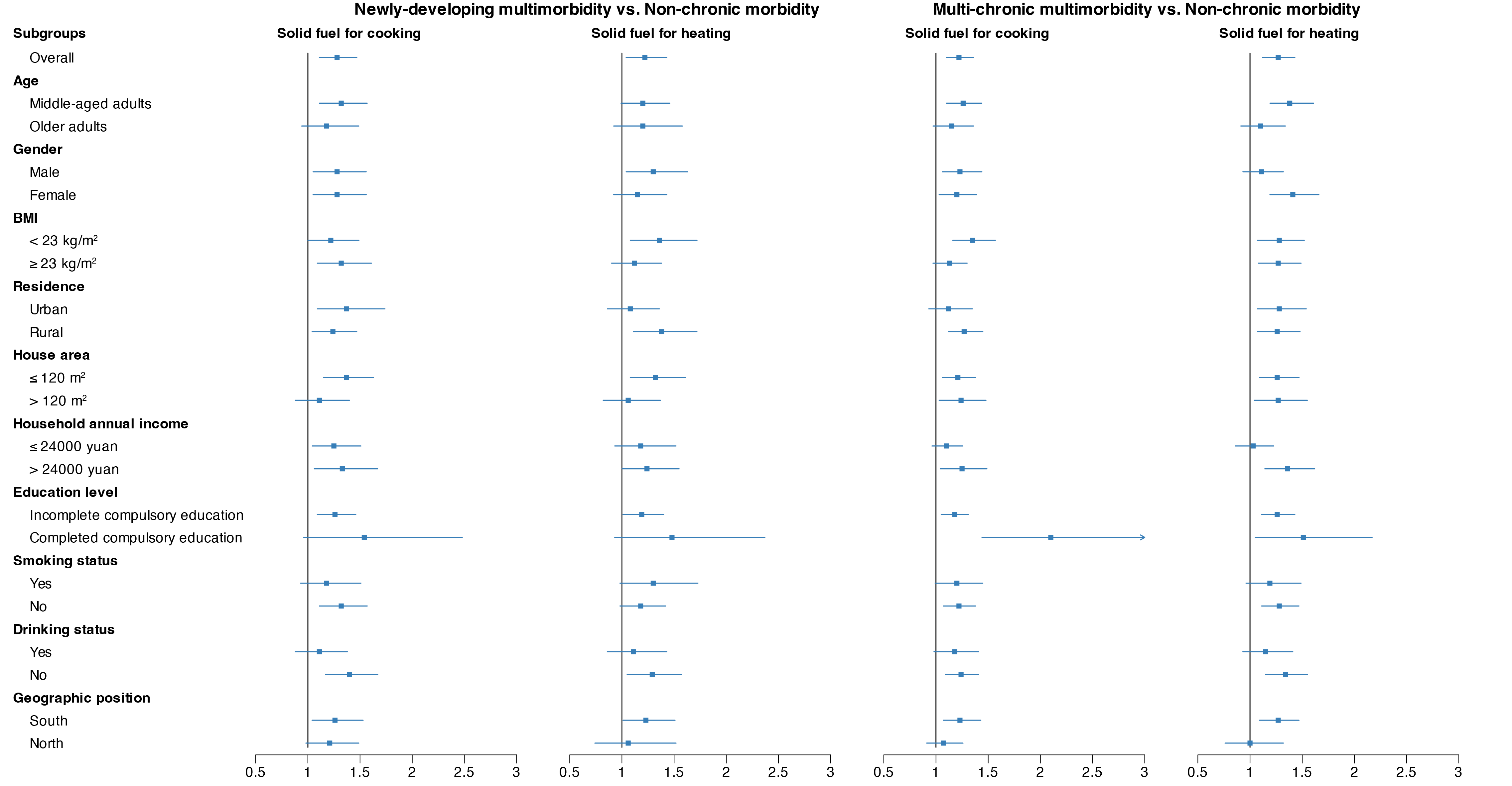
**

**Figure S3.** The association between different household fuel use and multimorbidity trajectories after stratified

All the models were adjusted by baseline age, sex, residence, education level, marriage status, smoking status, drinking status, BMI and PM_2.5_.

**
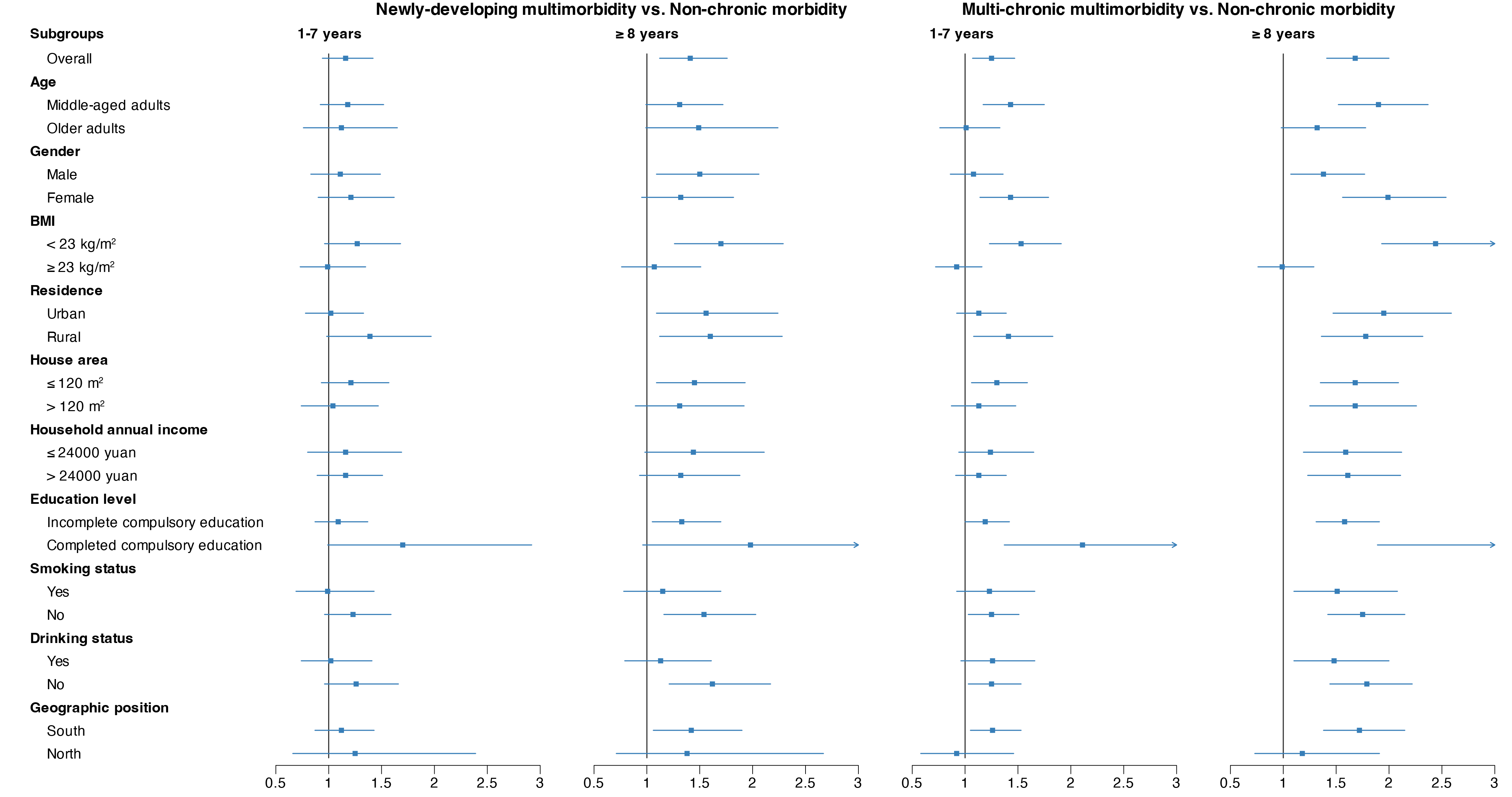
Figure S4.** The association between solid fuel using duration and multimorbidity trajectories after stratified

All the models were adjusted by baseline age, sex, residence, education level, marriage status, smoking status, drinking status, BMI and PM_2.5_.


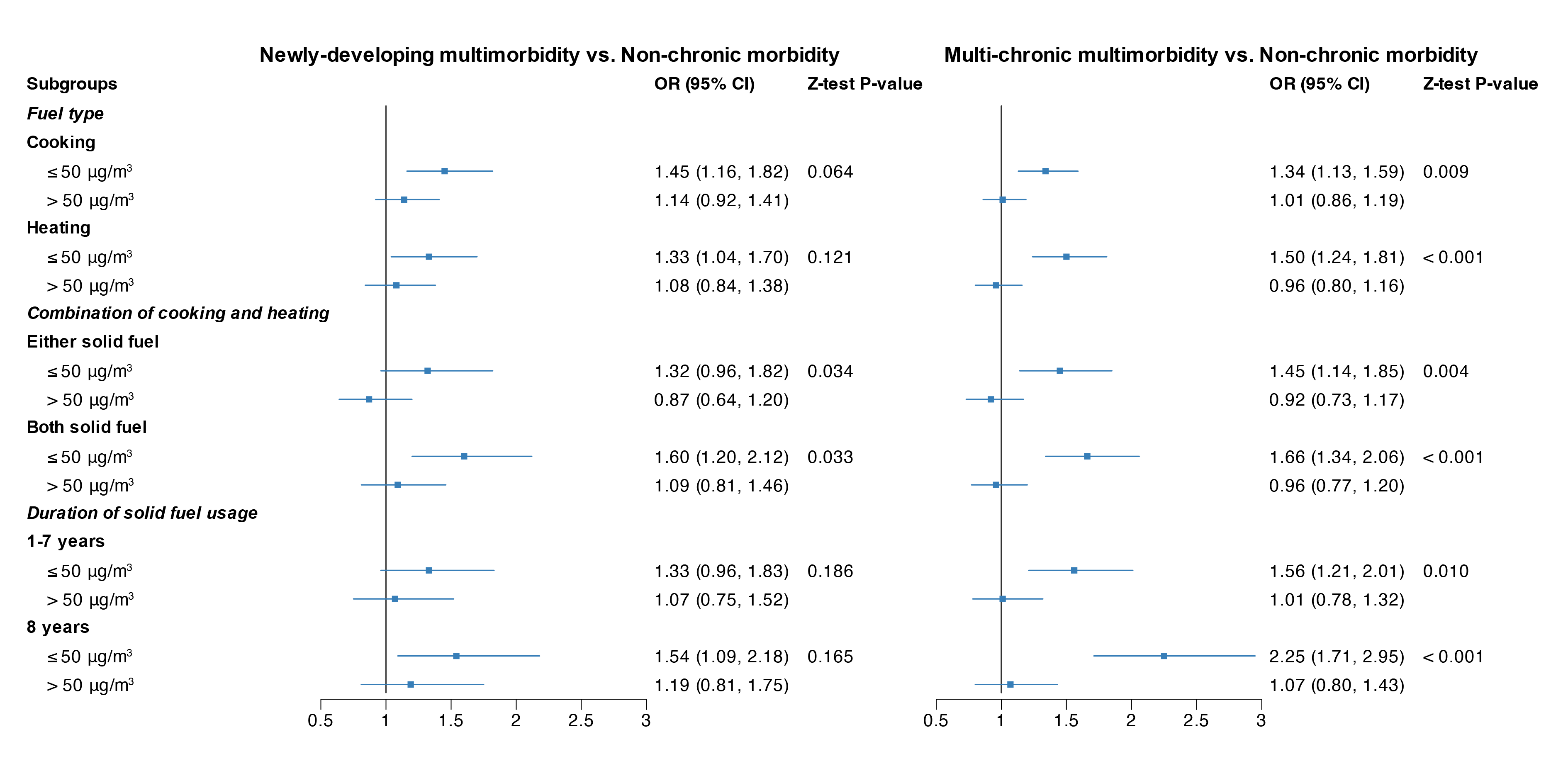


**Figure S5.** Stratified analysis by PM_2.5_ for the association of fuel types and duration of solid fuel usage with multimorbidity trajectories before imputation

Each line represented the risk of adverse multimorbidity trajectories associated with the usage of solid fuels within different PM_2.5_ subgroups; All the models were adjusted by baseline age, sex, residence, education level, marriage status, smoking status, drinking status, BMI and PM_2.5_.

**
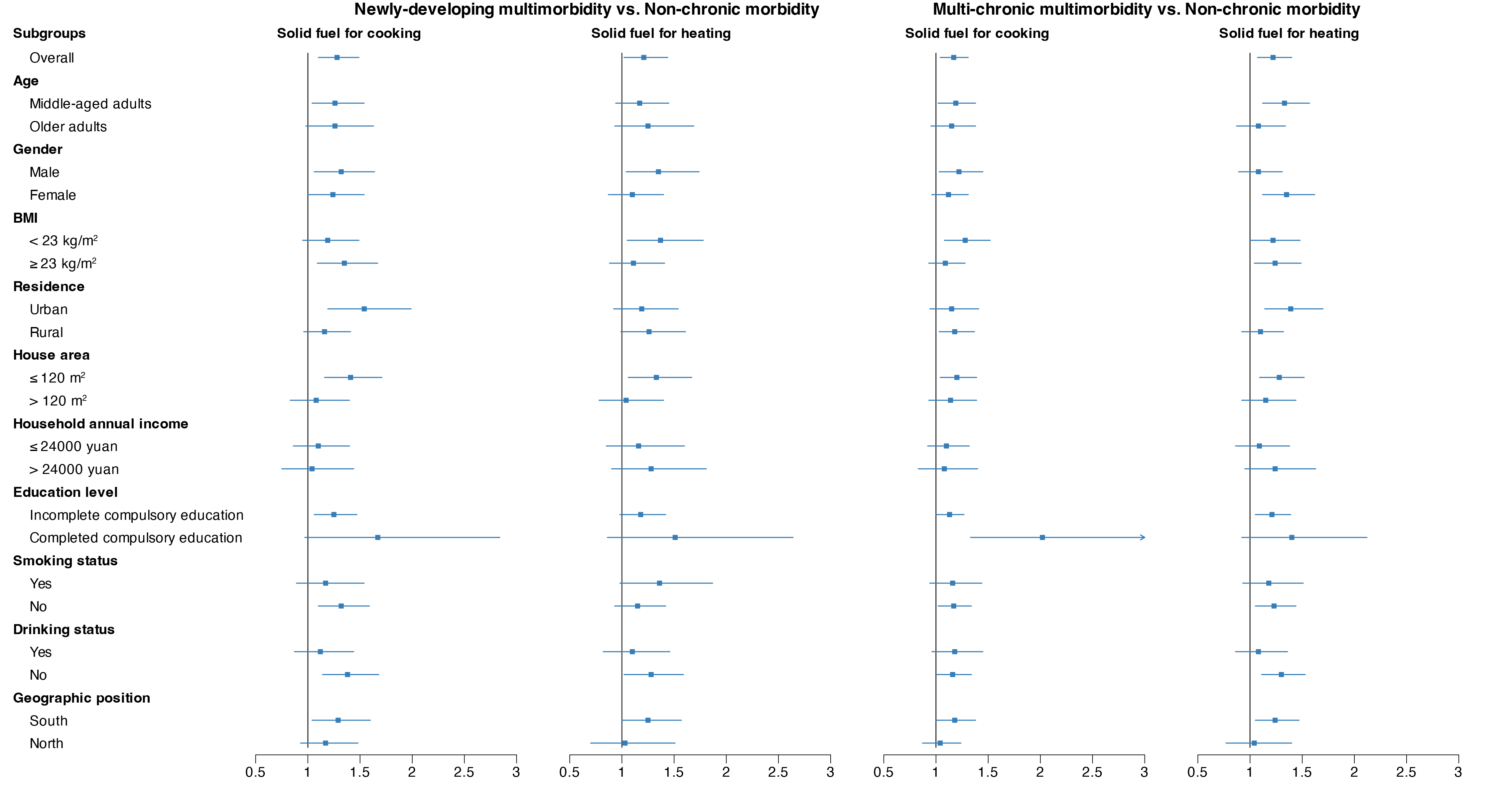
**

**Figure S6.** The association between different household fuel use and multimorbidity trajectories after stratified before imputation

All the models were adjusted by baseline age, sex, residence, education level, marriage status, smoking status, drinking status, BMI and PM_2.5_.

**
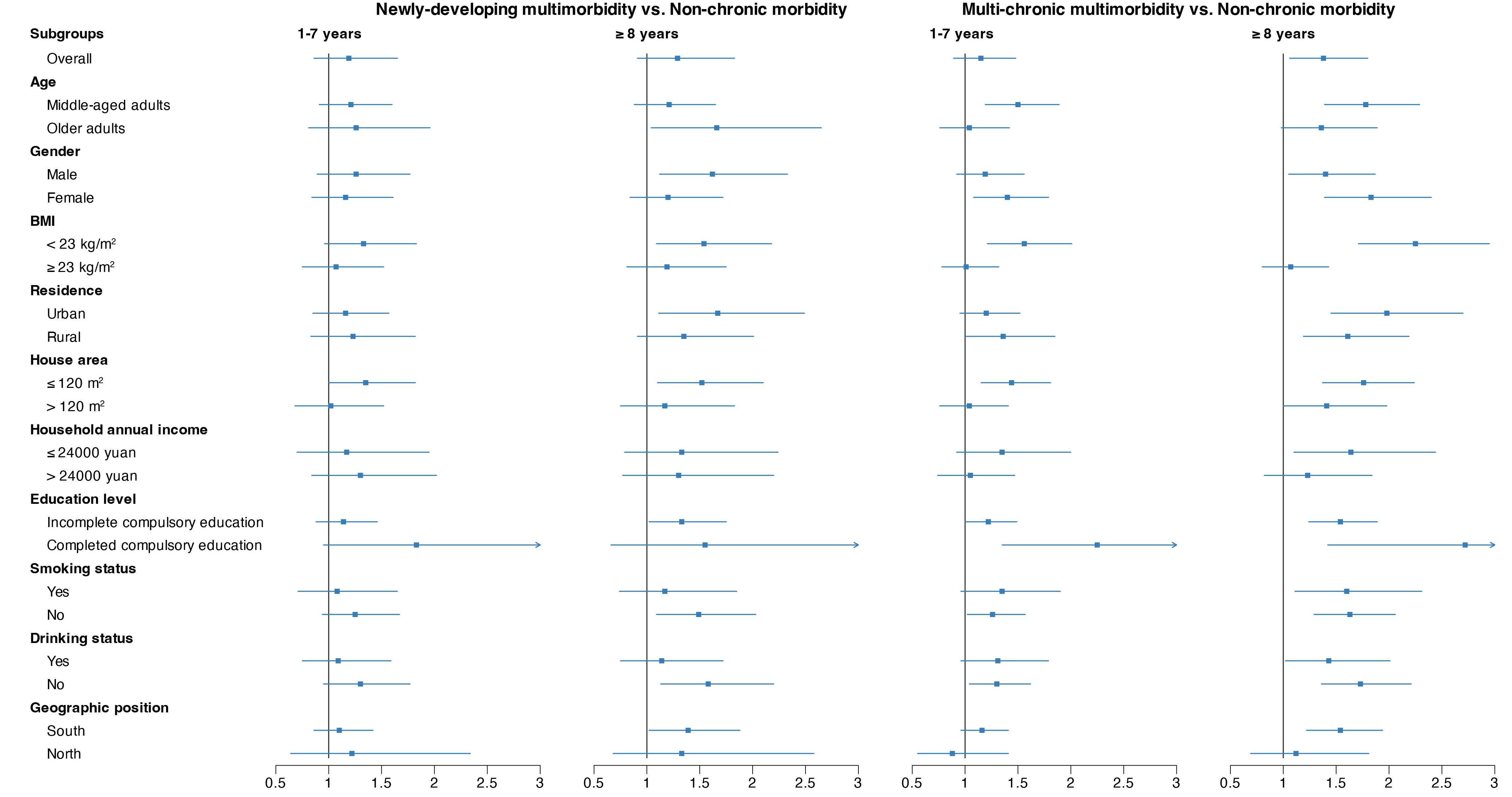
Figure S7.** The association between solid fuel using duration and multimorbidity trajectories after stratified before imputation

All the models were adjusted by baseline age, sex, residence, education level, marriage status, smoking status, drinking status, BMI and PM_2.5_.
